# Adulterated Boots

**Published:** 1896-01-04

**Authors:** 


### EDITOR'S LETTER-BOX.

[Our correspondents are reminded that prolixity is a great bar to publication, and that brevity of style and conciseness of statement greatly facilitate early insertion.]

### ADULTERATED BOOTS.

MR. F. PASSMORE, 11, Almeida Street, Islington, writes: A copy of your paper for November 16th last has just reached my hands, and I need hardly say that I have been much interested in its contents, and most particularly so with a note on the dangers of adulterated boots. I am not interested in the boot and shoe trade, but since the Leather Fair at the Agricultural Hall last April I have paid definite attention to the manufacture and composition of our modern foot coverings. At the exhibition I learned that ordinary boots possessed three soles, the outer, the inner, and the middle. The first two being open to our inspection, we can in a measure protect ourselves against the use of inferior material, but with the hidden middle sole the case is different, and we are entirely at the mercy of the unscrupulous manufacturer, who, on economy bent, fills up the boot with a miscellaneous assortment of materials, including the commonest of felt, curriers' waste, strawboard, the mysterious "compo," and the equally mysterious leather board. These are all spongy as you describe them, and when once damped can never be thoroughly dried. The evils attending the wearing of boots made in this manner are too patent to require description, and your warning to avoid them was none too emphatic.

At the same time that I attended the Leather Fair I made acquaintance with an invention which, when adopted, completely obviates any possibility of wet rising from the ground into the boot, besides enjoying many other merits of special value. The invention consists of a preparation of asbestos wool compressed into thin sheets by hydraulic pressure. These sheets are then waterproofed on one side by a special solution and portions inserted into the boots as the middle soles. Asbestos is, perhaps, the greatest non-conductor of heat known to science, and its interpolation into the fabric of our boots has the effect of preventing the passage of heat between the body and the ground. In winter time the cold ground cannot extract our bodily heat, while in summer time the ground heat cannot rise through the boots into our system. The waterproofing also excludes the damp. Asbestos, being of a porous character, readily absorbs whatever excess of perspiration there may be, which is evenly evaporated when the boots are removed. Further, asbestos-lined boots cannot creak in wear, and they are, besides, many times more flexible than boots made in the ordinary manner. Lastly, asbestos being also a non-conductor of electricity, persons wearing these boots may walk over live electric wires without fear of danger. I am informed that the invention has been brought to the notice of the War Office authorities, who evidently think so well of it that two separate sets of samples (amounting to about 500 pairs), have been ordered from a Northampton firm for the purpose of experiment, so that there is every prospect of the army being in time furnished with the asbestos-lined boots. It may interest you to learn that over 250 of the very largest boot manufacturers in the kingdom have adopted the invention, and are now turning out boots with the asbestos middle sole in large quantities, so that there should be no difficulty in obtaining them.

The invention is that of a Dublin medical man. I may add, finally, that I have worn a pair of asbestos-lined boots constantly since last May, and in all descriptions of weather they fully answered the expectations formed of them. I enclose you a small sample of the lining.

